# Utilizing the Foreign Body Response to Grow Tissue Engineered Blood Vessels in Vivo

**DOI:** 10.1007/s12265-017-9731-7

**Published:** 2017-02-15

**Authors:** Wouter J. Geelhoed, Lorenzo Moroni, Joris I. Rotmans

**Affiliations:** 1 Department of Internal Medicine, Leiden University Medical Center, Leiden, The Netherlands; 2 Eindhoven Laboratory of Experimental Vascular Medicine, Leiden University Medical Center, Leiden, The Netherlands; 3 MERLN Institute for Technology Inspired Regenerative Medicine, Complex Tissue Regeneration, Maastricht University, Maastricht, The Netherlands

**Keywords:** Vascular tissue engineering, Tissue engineering, In vivo tissue engineering, Graft, Vascular graft, Vasculature, Vascular access, Animal models, Translational, Graft patency, Foreign body response

## Abstract

It is well known that the number of patients requiring a vascular grafts for use as vessel replacement in cardiovascular diseases, or as vascular access site for hemodialysis is ever increasing. The development of tissue engineered blood vessels (TEBV’s) is a promising method to meet this increasing demand vascular grafts, without having to rely on poorly performing synthetic options such as polytetrafluoroethylene (PTFE) or Dacron. The generation of in vivo TEBV’s involves utilizing the host reaction to an implanted biomaterial for the generation of completely autologous tissues. Essentially this approach to the development of TEBV’s makes use of the foreign body response to biomaterials for the construction of the entire vascular replacement tissue within the patient’s own body. In this review we will discuss the method of developing in vivo TEBV’s, and debate the approaches of several research groups that have implemented this method.

## Introduction

Globally there is an immense and ever increasing need for vascular grafts for use as vessel replacement in cardiovascular diseases (CVD), or as vascular access site for hemodialysis. It is widely known that the incidence of CVD is increasing, a trend expected to continue in the foreseeable future [1, 2]. This coincides with an increase in the number of end-stage renal disease (ESRD) patients requiring a vascular access (VA) site [3]. Ideally, autologous veins or arteries are used as grafts, as these are associated with superior patency [4, 5]. However, due to a relative lack of donors, previous harvesting, or the poor state of the patients own vessels, native arteries and veins are not available for grafting in a substantial portion of patients. In such cases prosthetic grafts offer a suitable alternative and are frequently utilized. However, the primary patency for these synthetic grafts is dismal, both as arterial bypass, and arteriovenous graft for hemodialysis [6]. This failure of synthetic vascular grafts is primarily due to intimal hyperplasia, thrombosis and infection [7, 8].

Tissue engineered blood vessels (TEBV’s) may be a promising alternative for patients requiring a vessel replacement or VA site. Numerous approaches to the development of tissue engineered grafts have been described, and extensively reviewed [9–11]. The majority of these approaches tend to involve complex in vitro preparation steps, decellularized constructs, or the incorporation of synthetic materials onto the TEBV. Ideally, a vascular replacement is made completely out of cellularized autologous tissue, thereby not causing any immune reaction, and retaining the ability to remodel in vivo.

**Fig. 1:**
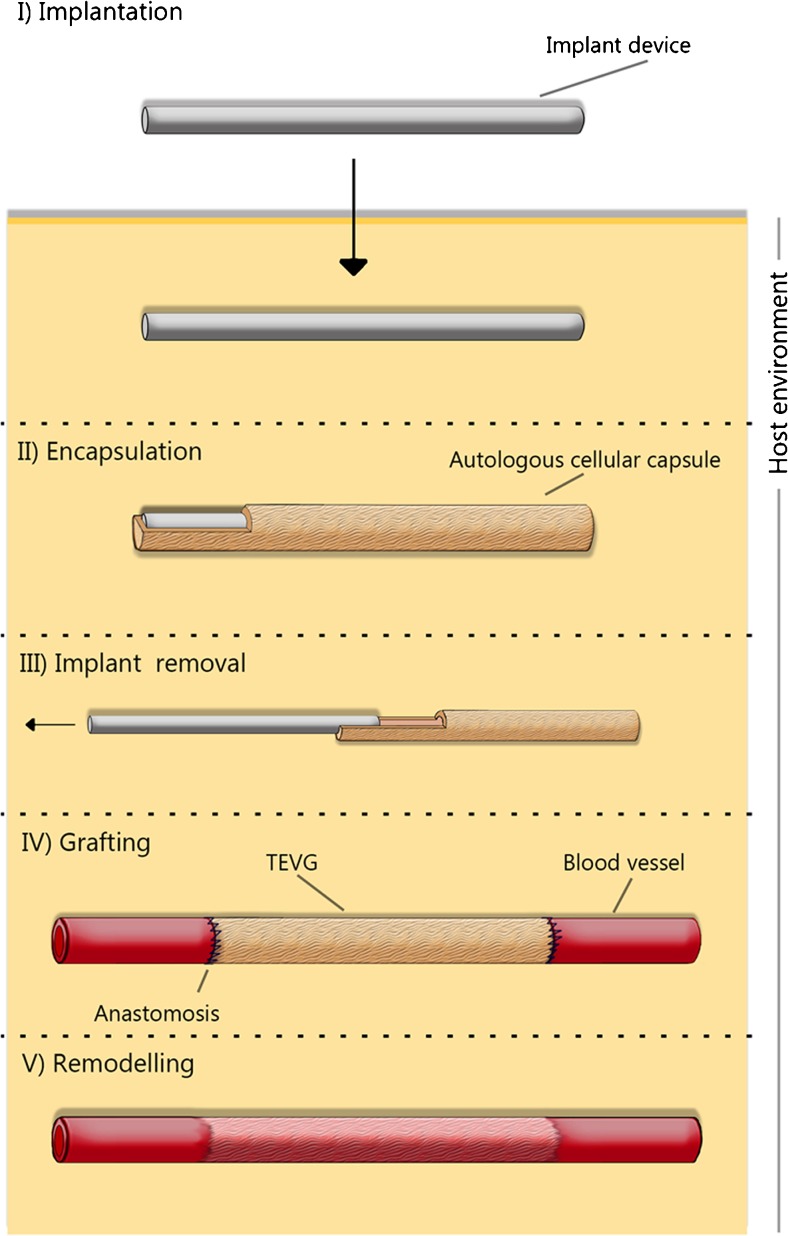
An overview of the in vivo based concept. I) a biomaterial is implanted in the host. II) The host environment acts as a bioreactor, leading to the encapsulation of the biomaterial with a cellularized fibrous tissue capsule. III) The implant device is removed, leaving only the tissue capsule. IV) The tissue capsule is grafted to the vasculature, creating a TEBV. V) Over time remodeling occurs, where the TEBV transdifferentiates to attain characteristics of a native blood vessel

In the present review, we will discuss approaches that utilize the host reaction to an implanted biomaterial for the generation of completely autologous TEBV’s in vivo. In other words, this approach to regenerative medicine and the development of TEBV’s aims to make use of the body’s foreign body response to biomaterials, and exploit the host environment as a bioreactor for the generation of new tissues, essentially allowing for the construction of the entire vascular graft within the patient’s body. Interestingly, most biomedical research concerning the foreign body response (FBR) is conducted with the aim of minimizing, or abolishing the cascade resulting in this host reaction, as propagation of the FBR is commonly associated with a decrease in implant functionality [12, 13 ]. Yet, by utilizing the FBR to generate tissue constructs in a controlled setting, various groups have developed methods to construct TEBV’s [14–17]. This involves an implantable biomaterial, which elicits a FBR to allow the growth of tissue around it (Figure 1.). TEBV’s made in this way would be non-toxic, elicit no immune response, and be free of pre-existing disease as the tissue is completely autologous, none of the initial foreign material remains in the body to propagate an immune response [18].

There are several requirements to which a TEBV’s must adhere in order to be considered as a promising vessel substitute. These factors must be taken into account when through the developing a TEBV. An overview of the requirements of TEBV’s is provided in Table 1.

**Table 1:** An overview of the requirements of a TEBV

***I)*** *the mechanical properties of the vessel must be sufficient to withstand the pressure caused by flow for extended periods of time without resulting in an aneurysm or bursting;*
***II)*** *the vessel should be sufficiently compliant to avoid a compliance mismatch, a known factor for graft failure* [19]*;*
***III)*** *the surgical suitability must be considered; the graft must have sufficient suture retention strength, and be easy to handle by the surgeon;*
***IV*** *) the vessel must be compatible with the host,* i.e. *not elicit an inflammatory reaction, be non-toxic, and non-carcinogenic;*
***V)*** *the vessel should be capable of remodeling to meet the demands of the vasculature;*
***VI)*** *the surface of the graft should not be prone to thrombus formation.*
***VII)*** *be produced in a large scale, cost effective manner, and available in various dimensions;*
***VIII)*** *show acceptable variation between batches.*

The concept of using the host as an in vivo bioreactor is not new. Already in 1961, Schillings et al. attempted to make autologous grafts by implanting stainless steel mesh cylinders subcutaneously for 4 months in 12 dogs. Five of these grafts failed due to thrombosis, technical errors and bleeding. However, the remaining 7 grafts showed an impressive patency of 3 years. Yet, it was Sparks who truly pioneered the application of autologous capsulated tissue as vascular grafts grown in vivo in the 1960’s, with the grafts being briefly applied in clinical applications [20, 21]. Dacron fabric grafts, covered by layers of fibrous tissue that formed as a result of the subcutaneous implantation of silicone rods, were clinically implemented as arterial bypass grafts in the late 1960’s. However, the application of this TEBV approach was reportedly low, as follow-up studies showed that there were various complications concerning the technique [22–25]. A main issue was the long incubation time that was required for the formation of the tissue around the implant, although this did not always yield a suitable graft. Thrombosis and stenotic occlusion of the graft was the main reason for late graft failure, while 20% of the grafts experienced aneurysm formation [23]. Little information is available on the mechanical properties of the Spark’s graft, which could give an indication of why aneurysm formation occurred. However, it is likely that a main downside of the Spark’s graft is the lack of sufficient mechanical properties (concluded by the high rate of aneurysm formation). Incorporation of the polymer into the tissue and the low cell density of the graft may be one of the explanations behind the aneurysm formation of the graft.

The pioneering work by Sparks illustrated that the host foreign body response could be tailored for in situ tissue engineering purposes. Although aneurysm formation limited its further clinical application, subsequent research groups aimed to fine tune and reinvigorate this approach for the generation of TEBV’s, by making use of the increasing knowledge of biomaterials and the dynamics of the FBR.

This review will discuss vascular tissue engineering (VTE) approaches that utilize the patient’s body as a bioreactor for the development of completely autologous TEBV’s. The context of these studies and their approach will be discussed. Furthermore, the complex process of clinical translation of and the use of appropriate animal models will be debated.

## The Foreign Body Response

In order to understand how the host environment can contribute to the formation of new tissues in response to an implanted biomaterial, it is important to understand the cells and pathways involved in the FBR. All biomaterials elicit a cellular and tissue response when implanted in vivo, known as the FBR [13, 26]. If a foreign body is small and superficial enough it will be extruded from the body. If the foreign body is too large to extrude, it will be encapsulated to ensure there is a safe barrier between it and the host. Initially, the implantation of a biomaterial causes mechanical damage to the vascularized connective tissue at the implant site. In this very early process of the FBR, blood material interactions caused by implantation result in the immediate adsorption of proteins onto the surface of the implant creating what is known as the provisional matrix [27].

The provisional matrix is composed of numerous bio-reactive agents, including fibronectin, complement components, albumin, and vitronectin. This makes it crucial in determining the activity, proliferation, migration, and differentiation of inflammatory and wound healing cells [26]. Notably, fibrinogen can absorb directly on the biomaterial surface, creating a dense fibrin network, which sequentially promotes leukocyte adhesion [28, 29]. This presence of fibrinogen is vital, as it has been shown that mice with depleted fibrinogen were unable to initiate an inflammatory response to implanted biomaterials [30]. Complement factors can spontaneously adsorb to the biomaterial, which can lead to the activation of the alternative complement pathway [31–33]. Due to the mechanical nature of the formation of the provisional matrix, its composition can vary greatly depending on the implant location, and surface properties of the implant [27]. Furthermore, variations in protein adsorption occur due to what is known as the Vroman effect, which describes a competitive protein exchange on biomaterial surfaces, i.e. the competitive displacement of adsorbed proteins by other proteins with stronger binding affinities [34].

Following the formation of the provisional matrix, the initiation of the acute inflammatory response is the next phase of the FBR, characterized by the infiltration of neutrophils. In this phase the wound site is cleaned, vessels dilate and blood flow to the injury site increases. A variety of cytokines and growth factors are released, monocytes infiltrate the implantation site and begin to differentiate to macrophages [26]. It has been shown that histamine inhibition significantly decreased phagocyte recruitment, elucidating the importance of mast cells and histamine in the acute FBR [35].

If the inflammatory stimulus persists, in case the biomaterial is not removed; the inflammatory response enters a chronic phase. Normally, the acute phase lasts from several hours to several days; chronic inflammation generally lasts no longer than two weeks as long as the inflammatory stimulus does not persist any longer [26]. Yet, the extent of damage that occurs at the implant site is vital in determining the length and severity of the acute, and the chronic inflammatory phases. This phase is characterized by monocyte infiltration, macrophage activation and angiogenesis of the site of tissue injury [26]. Angiogenesis is essential to support the wound healing process with a supply of nutrients. Following an early angiogenic pulse caused by fibrin [36], histamine [37], and VEGF (released by platelets), the angiogenic process is later maintained by hypoxic macrophages and fibroblasts in the new tissue [38].

**Fig. 2:**
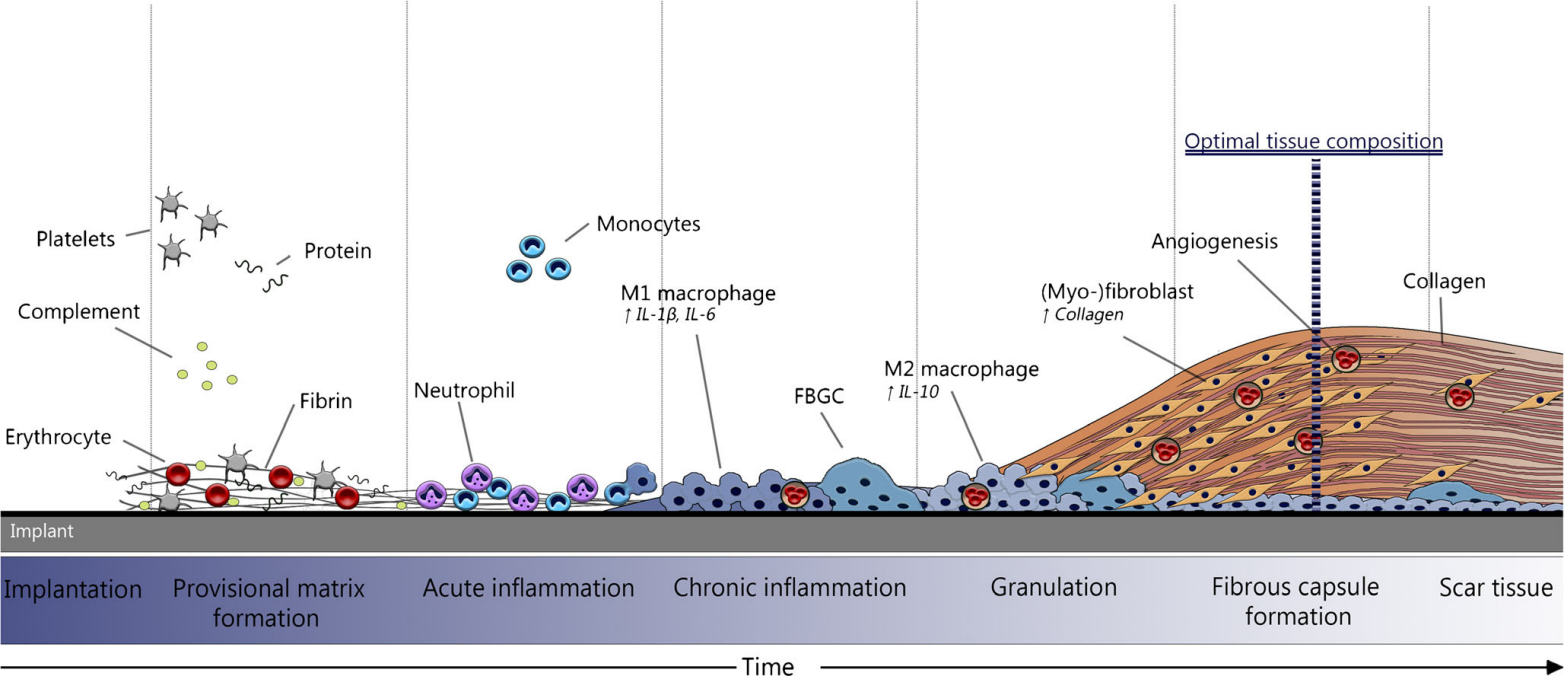
An overview of the foreign body response. Following implantation a provisional matrix immediately forms around the implant. Following provisional matrix formation acute inflammation is mainly characterized by the presence of neutrophil, and some monocyte infiltration and differentiation. Chronic inflammation is characterized by the infiltration of monocytes that differentiate to macrophages, and neovascularization. Fibroblasts then proliferate and begin to produce ECM components including collagen. A fibrous capsule forms composed out of a (myo-)fibroblasts, ECM components (mainly collagen), and a one- to two-layer of macrophages. Over time scar tissue forms mainly composed of ECM and collagen, with less fibroblasts. The optimal tissue composition for use as TEBV is a thick cellularized fibrous capsule, which is collagen rich with relatively few inflammatory cells

Monocyte and macrophage recruitment to the wound site is driven by numerous chemoattractants such as IL-1β, IL-4, TNF-a, and CCL2, which facilitate proliferation, and the extravasation of leucocytes [39–41]. Macrophages have been shown to initially secrete IL- β, and IL-6, and eventually express more IL-10 as time progressed, indicating a phenotypic shift from a pro-, to an anti-inflammatory state [42]. If the foreign body is too large to be phagocytize activated macrophages fuse together to form multinucleated foreign body giant cells (FBGC’s), a characteristic feature of chronic inflammation. Vitronectin, commonly present in the provisional matrix has been shown to support macrophage adhesion, and foreign body giant cell formation [43]. If the FBGC’s are unable to remove the foreign body, the process of encapsulation is initiated by surrounding the implant with a dense collagen matrix [44]. Macrophages and FBGC’s stimulate fibroblasts to proliferate and overproduce components of the extracellular matrix (ECM), including collagen, by releasing TGF-β, IL-4, IL10, IL13, TNF-a, and IL-1 [45, 46]. It should be noted that TGF-β is regarded as the most potent inducer of the ECM formation [38]. Granulation tissue is characterized by the immigration of fibroblasts, angiogenesis in the newly developed tissue, and the presence of a layer of macrophages or FBGC’s lining the implant. If the stimulus is not resolved, a thick fibrous capsule ultimately forms that is very rich in collagen, (myo-) fibroblasts, and one or two layers macropaghes and FBCG’s [26, 38]. It is proposed that if the inflammatory stimulus remains for multiple months, the tissue becomes less cellularized, ECM rich scar tissue as was seen in the Sparks’ graft. An overview of the FBR for biomaterials aimed at the generation of in vivo autologous TEBV’s is provided in Figure 2

## Tailoring the FBR

The host FBR to an implant is a complex reaction, where host features, implant characteristics, and implantation duration all contribute to the response generated. However, in its complexity it is clear that many factors can influence the host response to a biomaterial. Regardless, the host environment and surrounding tissue are an interesting cellular source for in vivo tissue regeneration strategies [47, 48]. Since the application of the Spark’s graft, much research has been done on the FBR and how it could be modulated to provide a cellular source for the construction of new tissue structures in vivo.

**Firstly**, biomaterial characteristics are critical in defining the FBR response that is generated. The chemical composition, hydrophilicity, topography, and coating of the biomaterial can critically influence the initial cell-material interactions that occur [49–51]. Therefore, by altering the composition of the biomaterial the resulting foreign body response can be modulated. For example, if the composition and adherence of the provisional matrix is altered, it can impact the following cellular reaction [52]. Moreover, macrophage adherence is known to be essential in driving the FBR [26]. If a material can alter macrophage adherence, this will also greatly influence the resulting FBR. For example, solvent etching and gas plasma treatment is known to affect cell attachment to biomaterial surfaces [53, 54]. **Secondly**, the length of implantation period is vital in determining the tissue structure that is developed. Very early in the foreign body response, the tissue will be largely composed of neutrophils, have a highly inflammatory profile, and low collagen content (and therefore poor mechanical properties). Yet, a too long incubation step (several months) will result in largely acellular tissue unable to adequately remodel in the vasculature potentially leading to aneurysm formation, as was seen in the Sparks graft. **Thirdly**, implantation location is vital in determining the FBR to a biomaterial. Fibroblasts differ at varying anatomical sites, and with disease processes [55]. Moreover, fibroblasts from separate tissues differ in their production of matrix metalloproteinases (MMP’s), the production of collagen, and proliferation, meaning that the degree of encapsulation is likely to vary between tissues [56].

## Overview of Approaches for in Situ Vascular Tissue Engineering

Since the application of the Sparks graft in the 1960’s, the group of Campbell was the first to reconsider the utilization of the body as a bioreactor for the generation of new tissue, by using the peritoneal cavity as implant location. The motivation behind this approach is that besides showing a sufficient FBR to the implanted material, mesothelial cells can be recruited to the tissue capsule. Mesothelial cells and endothelial cells have been shown to have various similarities, including a non-thrombotic tendency [57].

In both rat and rabbit models, a piece of silastic (an inert silicone elastomer) tubing was implanted in the peritoneal cavity. Two weeks after implantation the implants were harvested and the silastic tubing removed from surrounding tissue capsule. The resulting tissue capsule was covered by layers of myofibroblast, and a single layer of mesothelial cells. Next, the tissue capsules were reverted, so that the outer mesothelial layer of the capsule lined the lumen during grafting. The tissue capsules were implanted as arterial interposition, left in place for a maximum of 4 months. Ultimately, the TEBV’s showed an overall patency of 67% in rats, and 70% in rabbits. Prior to grafting, the granulation tissue was shown to be rich in β-actin, and desmin indicating the contractile capacity of the cells. However, low levels of smooth muscle myosin heavy chain (a marker for smooth muscle cells (SMC’s)) were observed in the capsules. One month after implantation, arterial levels of myosin heavy chain were reported, indicating a phenotypic shift of the myofibroblasts to a SMC-like cell. Furthermore, 1 month after implantation the presence of an internal elastic lamella was observed. The TEBV was shown to respond positively when treated with contractile and relaxing agents. However, only 50% of the implants produced suitable TEBV’s, occasionally producing no usable tissues in an animal, a notable limitation of this method [58].

Subsequently, the approach was attempted in 15 mongrel dogs, where various types of implant materials were assessed for their suitability as TEBV. Some of the implants were foreseen of an external mesh. 3–3.5 mm in diameter TEBV’s were implanted in either the peritoneal or pleural cavity for 3 weeks, allowing the formation of tissue capsules around the implants, and then grafted as arterial interposition in the femoral artery. In this model the TEBV’s were not everted prior to grafting. The mesh implants produced usable TEBV’s in all cases, yet had a poorer patency of 60% between 3 and 6.5 months. Only half of the implants without a mesh produced usable TEBV’s, yet had a patency of 83% between 3 and 6.5 months. The uncoated TEBV’s showed an impressive remodeling of its cellular composition. The myofibroblasts appeared to undergo a phenotypic switch to SMC like cells, and endothelization of the TEBV was observed as well. Furthermore, the TEBV had an adequate burst pressure of 2500 mmHg, allowing safe implantation into the vasculature [59].

More recently, the method was improved by allowing the TEBV’s to be generated under pulsatile conditions, significantly improving the mechanical properties of the vessels. The application of sheer stress has been shown to promote ECM protein production [60].

Clearly, the group of Campbell had shown the potential of a TEBV formed by the FBR within a host organism. A major advantage of the technique being everting the TEBV’s to expose mesothelial cells before grafting, whereas the invasive peritoneal implantation of the silastic tubing, comprises a relative disadvantage of this technique when compared to subcutaneous approaches that are discussed below.

Following the example of the group of Peirce et al., who in 1953 attempted to make vessel constructs out of aortic collagen, Tsukagoshi et al. attempted to create autologous TEBV’s using subcutaneously implanted silicone surrounded by a layer of fascia [61]. A major advantage of a subcutaneous implant location is the rapid regenerative capacity of the skin [62], which could lead to rapid development of tissue surrounding an implant. Besides only a silicone implant, a biological component from the host was added to the biomaterial in order to reinforce the TEBV. A 10x40mm segment of fascia from the dorsum and medial thigh of 15 rabbits was removed, wrapped around silicone tubes, and implanted in subcutaneous pockets. Four weeks later, the tubes were removed from the body and the silicone tube was removed from the fibrous capsule composed of fascia and a fibrocollagen mesh. The exposed collagen promoted platelet adhesion and thrombus formation [63]. A lumen composed entirely of collagen could therefore be highly thrombogenic. It was then interposed into the femoral artery of the rabbits as an end-to-end graft. Patency rates of 80% were reported at 5 and 8 weeks, and re-endothelialization of the TEBV was shown to occur. No aneurysm formation was reported, indicating that the method provided adequate mechanical strength. However, intimal hyperplasia was reported at both ends of the TEBV of 70% of the lumen, yet not in the center of the TEBV. This would indicate an increased proliferation of cells near both anastomoses likely as a result of the turbulent flow [16].

Another interesting approach has been developed by the group of Nakayama, who aims to create TEBV with a silicone implant. They have termed the fibrous tissue capsule that grows around their silicone implant ‘biotubes’. In their first report of the biotube they reported well-formed grafts a bursting pressure of at least 200 mmHg [64]. Subcutaneous implantation is advantageous due to the large population of dermal fibroblasts, which could promote the formation of new tissue.

In their first grafting study in rabbits, an 82% patency rate of the biotubes was reported at 12 weeks. Again, as the tissue capsules are largely composed of myofibroblasts and collagen. To reduce the risk of thrombosis, the potent anticoagulant Argatroban was administered to the fibrous capsule prior to grafting. Argatroban is commonly used in patients with heparin induced thrombocytopenia (HIT) requiring an antithrombotic therapy [65]. The tissue capsule around the biotube was composed of fibroblasts, whereas after grafting circumferential collagen, myofibroblasts and possible SMC were reported [66]. In a subsequent study, the TEBV remained patent for an impressive 2 years in one animal, and showed signs of both elastin formation and endothelialization [67]. The extremely limited sample size does fundamentally limit the conclusions that can be drawn. The concept appeared to be somewhat less successful in a rat model, where a patency of 67% at 12 weeks was reported. Again, elastin formation and endothelialization of the TEBV was reported. Several creative improvements to stimulate tissue growth were implemented on the implants, such as the addition of nicotine, optical stimulation using LED’s, and the addition of eosin-Y, which were all shown to stimulate fibrous capsule formation [68–70].

In a recent study the acute phase patency of a new biotube type, designed around a silicone cage as appose to a plain rod, was compared to the original silicone rod mediated biotube design in 6 beagle dogs. Following a 4 week implantation in the dorsal subcutaneous pocket, the silicone implant was removed and the resulting tissue capsule was grafted into the femoral artery. The new biotube design was shown to have a burst pressure of 1825 mmHg, compared to 944 mmHg for the original biotube [15]. At 7 days the acute patency was observed by means of angiography. The new biotube design had a 100% patency, compared to a patency of 33% for the original biotube [15]. This research again underlines the potential of the in vivo bioreactor approach.

Our group has also attempted to generate autologous grafts in situ by focusing on the surface characteristics implanted biomaterials. It is known that the surface characteristics of an implanted biomaterial are key in driving the FBR and fibrous capsule formation [18, 51]. Ultimately, we aim to develop a TEBV to be used as vascular access site for hemodialysis. Our reasoning is that, due to the growing number of patients requiring hemodialysis treatment and limited vascular access options, TEBV’s could fundamentally improve this huge clinical problem for hemodialysis patients.

In a study in 15 rats, various surface modifications of a few materials were assessed in their effectiveness in propagating an encapsulating response. These included gas plasma treatments, collagen I and TGF-B coating, and chloroform etching. While the thickest tissue capsules were produced using TGF-B and collagen coating, this tissue was less uniform and had a low cell density. It was shown that the copolymer poly(ethylene oxide terephthalate/polybutylene terephthalate) (PEOT/PBT) which was chloroform etched provided the most ideal tissue; providing a thick collagen layer with a high cell density of circumferentially aligned myofibroblasts and initial signs of elastin formation. It was shown that chloroform etching increased the surface roughness and oxygen content of the polymer, resulting in an enhanced cell adhesion [51].

Sequentially, the method was assessed in a porcine model. Chloroform etched rods were implanted subcutaneously in the abdomen of 4 pigs. Four weeks later the polymer implants were removed and two tissue capsules were implanted bilaterally as carotid interposition grafts. A 1 week patency of 100%, and 4 week patency of 88% was reported. Prior to grafting, the tissue capsules were shown to be largely composed of collagen, glycosaminoglycans, fibroblasts, and some myofibroblasts. Directly lining the lumen leucocytes were observed, with hardly any FBGC’s. After grafting an increase in luminal diameter was shown, where the luminal side of the tissue capsule was covered by a monolayer of endothelial cells. The initial leucocytes lining the lumen was no longer present, most likely due to hemodynamic factors. The remaining cells in the tissue were mainly SMC like cells. The potential of modifying the surface characteristics of a biomaterial to modulate the FBR after implantation was shown to be a viable method of producing a cell rich TEBV. Clearly, the FBR can be steered to generate tissues with for varying purposes, including TEBV generation.

An overview of the animal studies carried out by the groups discussed above, in which TEBV’s were grafted into the vasculature is provided in Table 2.

**Table 2:** An overview of studies utilizing the host as bioreactor for TEBV generation

Implant characteristics	Implant location	Additional treatment	# grafted	Length	Model	Grafting technique	Patency inspection	Patency	Time	Components reported n TC	Components reported after grafting	Burst pressure	Comments	Group
Silastic	Peritoneal cavity	Evert tissue capsule	30	10 mm	Wistar rats	Abdominal aorta end-to-end	Direct inspection	67%	2–6 months	Commonly seen macrophages, myofibroblasts, connective tissue, mesothelial cells.	α-SMA (+), myosin (+) SMC like cells.	-	Mesothelium remained after graftin	Campbell et al. [58]
Silastic	Peritoneal cavity	Evert tissue capsule	20	20 mm	New Zealand White rabbits	Abdominal aorta end-to-end	Direct inspection	70%	2–6 months	Commonly seen macrophages, myofibroblasts, connective tissue, mesothelial cells.	α-SMA (+), myosin (+) SMC like cells.	-	Mesothelium remained after graftin	Campbell et al. [58]
Numerous	Peritoneal cavity	Tubing type / mesh application	11	50–70 mm	Mongrel dogs	Femoral artery end-to-end	Clinical examination, doppler ultrasound scanning	No mesh 83% Mesh 60%	Between 3 and 6.5 months Between 3 and 6.5 months	α-SMA (+), vim (+), des (−) myofibrobalsts, some macrophages.	Myosin (+) SMC like cells. Monolayer of endothelium.	2500 mmHg,	Mesh improved encapsulation, but decreased patency	Chue et al. [59]
Silicone	Dorsum - subcutaneous	Argatroban	10	20 mm	Japanese white rabbits	Common carotid artery - end-to-end	-	75% 100% 75%	6 weeks 2 weeks 12 weeks	Predominant fibroblasts and collagen.	Circumfirential collagen, α-SMA (+) (myo-) fibroblast or SMC. Monolayer of endothelium. Few elastic fibers.	~1000 mmHG	Argatrobanloading to decrease thrombogenicity	Watanabe et al. [66]
Silicone	Dorsum - subcutaneous	Argatroban	1	30 mm	Japanese white rabbits	Common carotid artery - end-to-end	-	100%	26 months	Predominant fibroblasts and collagen.	Circumfirential collagen, α-SMA (+) (myo-) fibroblast or SMC. Monolayer of endothelium. Elastin lamina.	~1000 mmHG	Extremely long patency yet only one animal	Watanabe et al. [67]
Silicone	Dorsum - subcutaneous	Argatroban	6	20 mm	Female wistar	Abdominal aorta end-to-end	Angiography	67%	12 weeks	Collagen rich tissue.	Circumfirential collagen, aSMA (+) (myo-) fibroblast or SMC. Monolayer of endothelium. Elastin like mesh seen.	1085 mmHg	Introduction sheeth used Interesting presence of elastin	Yamanami et al. [71]
Type C – mold system	Dorsum - subcutaneous	Argatroban	6	10 mm	Female Beagles	Femoral artery end-to-end	ultrasonography	Original: biotube 33% Type C: 100%	7 days	Fibroblasts and collagen.	Elastin present. Thick, collagen rich, elastin present.	Original: biotube 944 mmHg Type C: 1825 mmHg	Improvement over original design	Furukoshi et al. [15]
Silicone, fascia	Medial thigh - subcutaneous	Fascia covered	15	10 mm	Female Japanese white rabbits	Femoral artery end-to-end	Milking test	60% 80% 80%	2 weeks 5 weeks 8 weeks	-	Fibroblast, endothelium ingrowth.	-	Interesting concept to incorporate dorsal fascia	Tsukagoshi et al. [16]
PEOT/PBT 55/45	Abdomen - subcutaneous	Chloroform etching	8	80 mm	Female landrace pigs	Carotid artery bilateral, end-to-end	Angiography	88%	4 weeks	(myo-)fibroblasts, ECM, some luminal macrophages.	Predominantly desmin (+) SMC like cells, no leukocytes, Monolayer of endothelium, some elastin positivity.	5211,04 mmHg (derived burst pressure)	Biomaterial surface modification to tailor FBR	Rothuizen et al. [14]

α- SMA, alpha-smooth muscle actin; SMC, smooth muscle cell; vim, vimentin; des, desmin; FBR, foreign body response

## Remodeling of TEBV after Implantation in the Circulation

Ideally, tissue engineered grafts mimic the composition of native vessels. These are composed of a thin layer of healthy endothelial cells, surrounded by layers of connective tissue, SMC’s, and elastic laminae depending on the location, diameter and function of the vessel. Grafts derived from fibrous capsule formation tend to be rich in collagen, myofibroblasts, and have one- to two-layers of macrophages directly surrounding the implant. Remodeling in circulation is an essential step in the maturation of these in vivo grown grafts to attain attributes that more accurately mimics native vessels.

A hallmark of a functional vessel is the presence of a healthy endothelial monolayer, as this reduces the thrombogenicity and promotes the homeostasis of a vessel [72, 73]. A major struggle for all tissue engineering approaches is the incorporation of a functional endothelium that can handle the arterial flow rates following a grafting procedure and remain in place long term [74]. Numerous attempts have been made to seed endothelial cells on both TEBV’s and vascular grafts prior to grafting, however this has proven to be deceptively complex [75]. Exposed collagen on the lumen of in vivo grafts provide a potentially thrombogenic surface shortly after insertion in the circulation. Campbell et al. already showed an elegant approach to this problem by lining the luminal side of the TEBV with autologous mesothelial cells [58]. Despite the difficulty of seeding an endothelium upon TEBV’s prior to grafting, all studies using the in vivo grafts have shown endothelialization of the lumen [14, 16, 59, 71]. Although, the function of endothelial progenitor cells is decreased in patients with chronic kidney disease [76]. Spontaneous endothelialization of decellularized TEBV’s has recently been demonstrated in ESRD patients [77]. It remains to be determined whether this endothelialization of the grafts results from circulating EPCs that adhere to the surface of the TEBV, or result from the migration of endothelial cells from neighboring vessels to the graft.

**Fig. 3:**
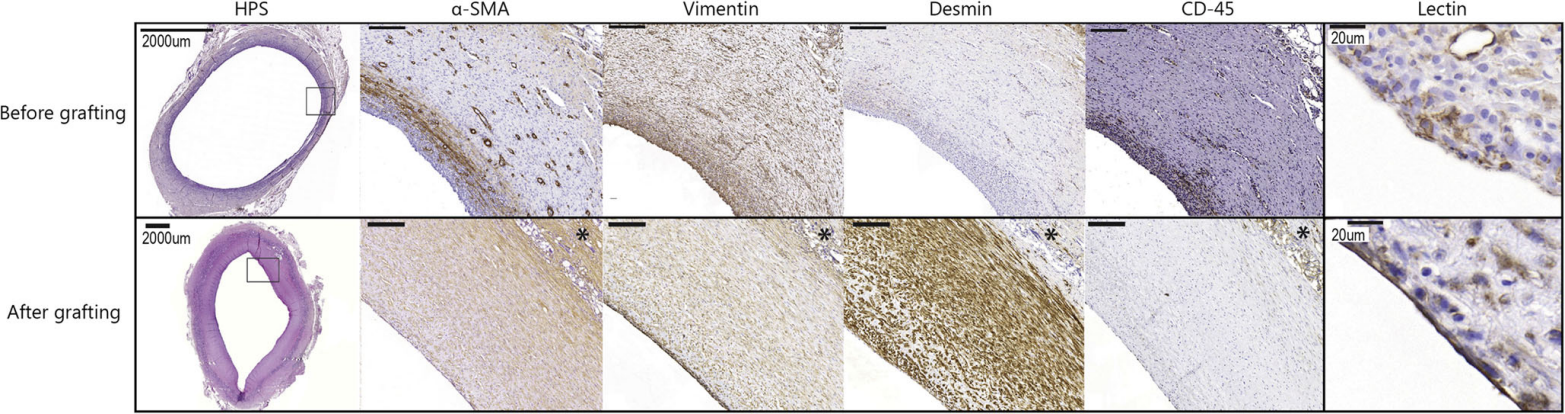
Adapted from Rothuizen et al. Showing an autologous tissue engineered blood vessel before, and after grafting. Before grafting α-SMA, vimentin positive (myo-)fibroblasts are present, with frequent CD-45 positive leucocytes and no endothelium (lectin negativity). After grafting the cells are α-SMA, desmin positive SMC like cells with no CD-45 positive cells and an endothelial monolayer [14]

Tissues grown as a result of the FBR are rich in fibroblasts and myofibroblasts, but not SMC’s. SMC’s are important in maintaining vascular homeostasis notably with regard to the vasoreactivity of vessels. Various studies have shown that after grafting, populations of (myo-)fibroblasts have either transdifferentiated to, of been replaced by SMC like cells [14, 58, 59, 66]. A histological overview of the differentiation of the tissue capsules towards blood vessel-like structures that we observed in our pig studies is provided in Figure 3. Moreover, Campbell et al. had reported an increased vasoactivity of the grafts after being implanted for several months [58, 59].

The structural integrity of a vessel is largely determined by the ECM, and is able to fundamentally modulate various aspects of cell biology in addition to its structural role [78, 79]. Here, collagen is the determining factor of the strength of the vessel [80]. Prior to grafting, TEBV’s that have grown as a result of a FBR will have a different ECM as well as cellular composition than native arteries. A breakdown of the ECM could therefore lead to diminished mechanical properties of the graft, and even aneurysm formation as was frequently seen in the Spark’s graft [20]. In circulation, collagen, which accounts for the mechanical strength of the ECM, can be broken down by matrix metalloproteases (MMP’s), in particular MMP-2 and MMP-9 [78]. This underlines the importance of a cellularized graft, which could allow the production of ECM components in vivo after it has been grafted into the circulation.

Another important component of the vessel wall is elastin, which is essential for vascular compliance [81]. Native elastin is immensely durable with a half-life of approximately 70 years [82], and relatively resistant to chemical and biological degradation [83, 84]. Thus far, the incorporation of elastin into TEBV’s which can remain functional for a long time, appeared to be extremely challenging [85, 86]. As elastin synthesis only rarely occurs in adult life, the incorporation of exogenous elastin fibers into the tissue might favor methods that depend on in vivo synthesis of elastin [85, 86]. Ideally, cells within the in vivo TEBV could remodel and begin to produce elastin after grafting, although the process by which this occurs in this the setting of FBR mediated TEBV’s is not entirely understood. Nayakama et al. for example, had reported the presence of elastin after grafting of the biotubes [71]. However, it remains to be demonstrated if functional elastic fibers are formed which have a positive impact on vessel compliance. Recently, we showed that miRNA29 inhibition could be an attractive method for elastogensis, with a superior elastogenic potential when compared to IGF-1, TGF-β1, and minoxidil [87].

## Advantages and Disadvantages of in Situ Vascular Tissue Engineering

There are clear advantages to using the host environment as an in vivo bioreactor. Grafts are composed of entirely autologous tissue, meaning no immunological mismatch is possible and no prior infection is present. The approaches to creating an in vivo TEBV require relatively simple polymer implants as opposed to any type of in vitro fabricated vascular construct. This means that this method has the potential of being cost effective and widely available.

Clear disadvantages of this method of course also exist. The grafts are limited in their applicability for acute procedures, due to their biological incubation time that generally requires several weeks. When utilizing in situ engineered blood vessels, the length required for certain arterial bypass procedures may not be attainable. For instance, creating femoral-popliteal bypass grafts that cross the knee joint could be challenging, as this requires flexible implant material to allow bending during the growth of the TEBV. The state of the host environment can alter the response to an implant. Gender [88], age, and the presence of disease can alter the response to an implant, and therefore the composition of the generated tissue. End-stage renal failure can impact wound healing and impair bone marrow function [76, 89]. Therefore the applicability of the grafts in these patient populations must be assessed in detail. As seen in some of the methods discussed in this review, there is a potentially large biological variation that must be assessed and controlled before these techniques can be considered for clinical translation.

## Animal Models

Ideally, an animal model is cheap, quickly develops a desired pathology, is readily available and mimics vascular setting of humans as closely as possible (i.e. thrombogenicity, vessel size, immune response). Clearly no animal model exists that fulfills all these requirements. In the development of a medical device and its translation to the clinic, animal models are indispensable. However, due to fundamental differences in the molecular pathways and pathologies between animal models and humans, animal studies do not translate well to the clinical phases of development [90].

For studying molecular pathways, mice are an ideal model, due to the large number of research tools and knockout animals available. However their small size makes vascular grafting studies suboptimal. The slightly larger size of rats and rabbits make these more suitable for early proof of concept studies. Limitations here are the poor translation to the clinic, as well as the relative lack of knock-out animals compared to mice.

The vessels of larger animals are more representative of humans and are more appropriate to mimic human vascular conditions, as the size of the vasculature is determinant for the sheer stress exacted on the vessel wall. Numerous sheep and goat models have been described in vascular studies [91–93]. However, high variability in response to anticoagulant and antiplatelet therapy in these animals is known to occur and must be taken into account [94]. Dogs have comparable vasculature to humans, are easy to handle due to their familiarity with humans, and readily available. Yet, dogs have however been found to be hypercoagulant in an ex vivo analysis [95]. However, synthetic grafts have shown high patency rates in dogs, with little sign of intimal hyperplasia, raising the question if dogs are a stringent enough model for vascular applications [96]. The vasculature of non-human primates is most similar to humans, making it the most accurate animal model available. However, due to high costs, special housing requirements, and stringent ethical concerns, the use of this model is limited.

Arguably, the ‘aggressiveness’ of the vasculature is the most important factor in assessing the long-term patency of vascular grafts, with intimal hyperplasia being the most common cause of TEBV failure. Different animal models vary greatly in the speed at which they develop intimal hyperplasia [97]. One of the most commonly used models in cardiovascular research today is the pig model [97]. The responsive nature of the vasculature of the pig makes it ideal for short-term pathophysiology studies. However, it is known that pigs for develop stenotic lesions up to six times faster than humans [98], making them less appropriate, arguably too stringent, for long term patency studies.

## Clinical Perspective

There are factors to take into account in the translation of TEBV methods to a clinical setting. Firstly, a TEBV derived from the FBR would require an additional implantation procedure, besides the grafting of the TEBV into the vasculature. Aside from this additional intervention, the use of these TEBV’s may be somewhat surgically challenging, as the TEBV must first be removed from the implant before it can be grafted to the vasculature. The procedure would therefore require additional training by a surgeon to carry out properly. Following a surgical intervention, such as the implantation of a medical device, a patient may be given immune suppressants. Immune suppression may intervene with the FBR, and therefore TEBV development, which would need to be accounted for when considering FBR based TEBV methods in a clinical setting. Before a FBR mediated TEBV method is to be considered for a clinical setting, the variation between patients must be well known, and accounted for, to assure all TEBV’s meet all requirements set for vascular grafts. It is known that for example CKD [89] and diabetes [99] can impair wound healing. Therefore it is vital to show that the formation of the TEBV’s in these patient populations occurs as expected, and with acceptable variation between patients.

In a pioneering clinical trial, the group of Shinoka successfully employed TEBV’s, designed from bone marrow mononuclear cells, with no graft related mortality, indicating the potential of TEBV’s in a clinical setting [100]. In a more recent clinical trial, the group of Niklason employed human acellular vessels as vascular access in 60 patients with ESRD, showing a primary patency of 28%, and secondary patency of 89% at 12 months post implantation, underlining the potential of TEBV approaches as potential vessel replacement [77]. VA sites offer an interesting target for the clinical implementation of TEBV’s. The occlusion of an arterial bypass is potentially fatal, while the occlusion of an arteriovenous conduit may render the VA site unusable, but is less dangerous for the patient, minimizing the risk of the trial. As was also stated earlier, the current options for creating a functioning arteriovenous graft are poor. Implementing a TEBV as an arteriovenous graft can be seen as a stringent model to assess TEBV functionality and patency, due to the harsh hemodynamic conditions, and frequent stenosis seen in arteriovenous conduits. Until now no FBR mediated TEBV approach has been assessed in a clinical phase. Our group is expecting to initiate the first clinical phase of our FBR mediated TEBV’s as VA before 2018.

## Conclusion and Future Perspectives

In this review we have illustrated and summarized the potential of a body as a bioreactor for the generation of autologous tissue engineered blood vessels in vivo, including the mechanisms of the foreign body response that can result in new tissues, research groups that have attempted to utilize this approach, and the difficulties and limitations of developing such methods. The potential of autologous in vivo made grafts is clear through the promising pre-clinical studies that have been carried out. With a continued understanding of the FBR, and the common factors leading to graft failure, we foresee more fine-tuned approaches to the generation of TEBV’s will be assessed. The limitation of the approach has been elucidated, and must be overcome for the methods to ultimately be successful. The translation of not only these, but all vascular tissue engineering approaches remains difficult, and suitable animals models must be chosen to allow for successful clinical translation. In conclusion, autologous in vivo TEBV’s show great potential as cell rich vascular grafts capable of remodeling in the vasculature.
